# Detection of bilirubin by stripping voltammetry at the gelled anisole – aqueous electrified interface supported with a 3D printed pore

**DOI:** 10.1007/s00604-026-08130-3

**Published:** 2026-05-21

**Authors:** Karolina Marciniak, Konrad Rudnicki, Karolina Kowalewska, Grzegorz Kowalski, Michal Poltorak, Irena Walecka, Grégoire Herzog, Lukasz Poltorak

**Affiliations:** 1 Electrochemistry@Soft Interfaces Team, Department of Inorganic and Analytical Chemistry, Faculty of Chemistry, University of Lodz, Tamka 12, Lodz, 91-403 Poland; 2 University of Lodz Doctoral School of Exact and Natural Sciences, Jana Matejki 21/23, Lodz, 90-237 Poland; 3 University of Lodz, BioMedChem Doctoral School of University of Lodz and Lodz Institutes of Polish Academy of Sciences, Jana Matejki 21/23, Lodz, 90-237 Poland; 4 The National Institute of Medicine of the Ministry of the Interior and Administration, Woloska 137, Warsaw, 02-507 Poland; 5 Université de Lorraine, CNRS, LCPME, Nancy, F-54000 France

**Keywords:** Urine, Stripping voltammetry, Organic gel, Bilirubin, 3D printing

## Abstract

A method is reported for the detection of bilirubin its anionic form (BIL^2−^) by stripping voltammetry at the electrified liquid-liquid interface. In this respect, we have used the aqueous || anisole and aqueous || anisole-PVC gel-based interface as an electroanalytical platform to study interfacial ion transfer reactions of model ion (TPrA^+^) and BIL^2−^. The gelled organic phase was supported with a 3D printed pore having a few hundred µm in diameter that, upon contact with the aqueous solution, was polarized. Next, cyclic voltammetry and linear sweep voltammetry was used with or without the stripping step to study the electrochemical behavior of TPrA^+^ and BIL^2−^ at the aqueous || anisole and the aqueous || anisole-PVC gel-based eLLIs. BIL^2−^ detection was studied in spiked artificial and real urine samples given that this analyte should be absent in the healthy patients urine. We have found that anisole is a suitable solvent for organic phase gel preparation, it stabilizes the LLI within the pore and allows the BIL^2−^ detection with a LOD = 2.84 µM and sensitivity (for real and spiked urine sample) of 0.181 nA·µM^− 1^ (pore Ø = 330 μm). These findings further underline the utility of the sensors based on the ion transfer.

**Graphical Abstract:**

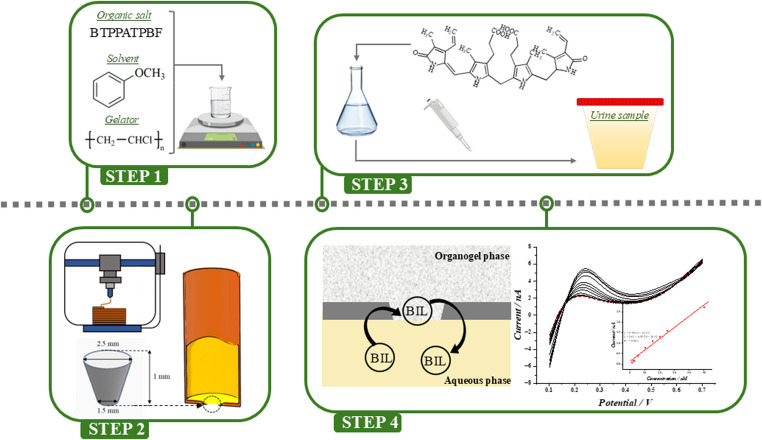

**Supplementary Information:**

The online version contains supplementary material available at 10.1007/s00604-026-08130-3.

## Introduction

Unlike traditional electrochemical systems that rely on redox reactions studied at the solid electrode surface, the interface between two immiscible electrolyte solutions (ITIES) is used to study interfacial charge transfer processes (e.g. ion or heterogeneous electron transfer) [1]. Since ITIES is defect-free down to the molecular level, can be renewed, and is self-healing, it holds some similarities to mercury electrodes [2, 3]. These properties ensure very high reproducibility and replicability of the experiments performed with ITIES by different teams worldwide. The adjustment of ITIES properties can be accomplished by interfacial modification (with e.g. nanomaterials, membranes, polymers, etc.) [4–6] and/or modulation of the composition of the aqueous and the organic phase. For example, by the dissolution of adequate ionophores or ligands in the organic phase, we can control the selective extraction of ions from the aqueous phase, which is crucial for applications in sensing, pharmaceutical analysis [7–9] or separation processes [10]. Moreover, ITIES can easily be miniaturized to micro– and, further to, nanometer dimensions. This is achieved with mechanical support using capillaries [11–14] patterned membranes with specific pore arrangements or intrinsically porous membranes [15–21]. When the ITIES surface area is diminished, diffusion layer profile transitioned from linear to hemispherical, ensuring higher mass transfer across the ITIES, which improved sensitivity. Another very interesting scientific direction relying on fabrication and miniaturization for electrochemical applications is the combination of microfluidics and ITIES to study the interfacial charge transfer process in dynamic conditions [22–24]. A straightforward miniaturization approach enables LLI to be positioned within small pores or apertures, yielding several benefits, such as improved stability, enhanced sensitivity, and lower sample (and other chemicals) volume requirements.

Typical detection limits of the unmodified, macroscopic ITIES-based system for small molecules such as inorganic ions or pharmaceuticals usually start from a few µM [25–27]. The limits of detection (LOD) can be improved via a gelation of the organic phase, increasing the mechanical stability of the ITIES and contributing to lowering the LODs down to a few nM [28–32]. The gelation of the organic phase allows the application of stripping voltammetry (SV) techniques since analytes may be concentrated at the ITIES. The preconcentration in the gelled organic phase is due to its higher viscosity, which lowers the diffusion coefficients of charged molecules and leads to their accumulation near the interface. Such methodology was recently applied to detect different molecules, such as perfluoroalkyl substances [33, 34] or small molecules like choline [35]. The organic gel phase is typically the mixture containing 10% w/v of a polymer (e. poly (vinyl chloride), PVC), a solution of organic phase electrolyte dissolved in an organic solvent (e.g. 1,2-dichloroethane, 2-nitrophenyl octyl ether, nitrobenzene, or fluoro-2’-nitrodiphenyl ether) [36–39]. Chlorinated solvents have been favored as organic solvent for electrochemical studies at the ITIES for their physicochemical properties: (a) high immiscibility with water; (b) sufficient dielectric constant assuring at least partial dissociation of the electrolyte into ions; (c) broad potential window assuming proper choice of background electrolytes. However, their use should be avoided due to their toxicity, persistence in the environment, and challenges associated with their safe disposal. Additionally, their carcinogenic potential and regulatory restrictions further limit their practical utility. In our study, anisole was used as a solvent for the organic electrolyte salt used to form ITIES. We decided to apply anisole due to (i) its lower toxicity compared to chlorinated solvents [40]; (ii) a high boiling point (154 °C), which is beneficial during the gel phase formation; (iii) lower disposal cost, again being beneficial from the environmental point of view [40]; and (iv) the possibility of polarizing the water || anisole interface [41].

The emergence of 3D printing (3DP), or additive manufacturing, has revolutionized the fabrication of labware by providing affordable and easily accessible tools for nearly real-time production. This technology offers high precision, rapid prototyping, and the ability to customize equipment on demand, making it a cost-effective alternative to traditional manufacturing methods, particularly for electrochemical sensing platform fabrication [42]. Most of the works describing the application of 3D printed platforms for electrochemical applications are focused on the production of electrodes (being part of sensing units or energy storage platforms), electrochemical cells, and microfluidic devices. Descriptions of manufacturing technologies and properties of printable materials (including synthetic and formulation protocols) can be found in a number of review publications that have been blooming over the last few years [43, 44]. Among many 3DP methods, Fused Deposition Modeling (FDM) stands out as a particularly accessible and versatile option. Yet, FDM has inherent limitations when it comes to fabricating small features like pores and channels. A significant challenge is its relatively low resolution in XY plane, which restricts the ability to produce the fine details essential for applications such as microfluidics. These limitations include (i) nozzle size, which is incompatible with highly viscous thermoplastic; (ii) step motor parameters; (iii) thermoplastic anisotropy; and (iv) difficulty in controlling the contact surface between deposited filament and underling support (previously deposited thermoplastic). Nevertheless, a number of elegant works pushed the limits of FDM-based printing to create electrochemical devices with features having micrometer dimensions. The first example demonstrates the use FDM-3DP to create an integrated platform for measuring the electric properties of liquids. This platform was successfully applied to measure the conductivity of aqueous KCl solutions, bottled water, and the permittivity of water-ethanol mixtures [45]. An interesting approach allowing the 3DP of microelectrodes was also reported by Helú et al.. by placing the metal or carbon micro wires/fibers in between insulating 3DP PETG layers during fabrication process [46]. Among the most significant advantages of the described methodology are the ability to control the size and geometry of features with a few tens of µm accuracy and the adaptability to produce structures with a wide range of thermoplastic materials. The availability of printable filaments made out of polyamides that are resistant to the action of organic solvents allowed the fabrication of platforms that can support electrified liquid/liquid interfaces within 3DP objects [14]. In our previous works, we have shown that (i) heroin detection can be done in the droplet (with a volume of up to 10 µL) placed in the printed aperture [41], or (ii) ephedrine can be quantified in the macroscopic four-electrode 3DP polyamide cell [47].

In this work, we have studied the electrochemical properties of the water || anisole and water || anisole-PVC gelled phase systems. The organic phase in the latter configuration was placed in a 3D printed capillary tube that supported the water || anisole-PVC gel-based LLI. We have performed a series of experiments to (i) define the utility of the anisole as the organic phase solvent for the chosen analyte detection and its compatibility with supports made out of polyamide; (ii) adjust the gel-phase formulation; (iii) optimize the miniaturization procedure; and (iv) use the developed platform for electroanalytical applications. The development of the electroanalytical detection protocol involved testing voltammetric methodologies, investigating the possibility of analyte preconcentration and stripping, and finally evaluating the electroanalytical parameters, such as sensitivity and detection limits. Initially, all experiments were performed with tetrapropylammonium cation (TPrA^+^) used as a model ion. Next, to display possible practical applications, we have applied our system to detect the anionic form of bilirubin (BIL^2−^) dissolved in the electrolyte solution, spiked artificial and real urine samples [48]. BIL^2−^ is considered a biomarker of liver function, specifically reflecting the liver’s ability to process and eliminate waste products. Elevated levels of BIL^2−^ in urine can indicate liver diseases, such as hepatitis, cirrhosis, or bile duct obstructions, and are crucial for the diagnosis and the monitoring of conditions related to impaired liver function [49, 50]. Early detection of BIL^2−^ in urine helps in the timely diagnosis and management of these disorders.

This work uniquely combines a safer, anisole-based gelled organic phase with a solvent-resistant 3D-printed polyamide capillary to create a miniaturized, stabilized LLI. To the best of our knowledge, this is the first demonstration of anisole-PVC gel in a 3DP support for ITIES electroanalysis, enabling stripping voltammetry–based preconcentration in sub-microliter volumes while avoiding chlorinated solvents. The platform is compatible with common 3DP materials, amenable to rapid prototyping, and is validated for a clinically relevant analyte (BIL in spiked and real urine), highlighting its practical potential for low-volume, safer electroanalytical sensing.

## Method and materials

### Chemicals

The electrolyte dissolved in the organic phase was synthesized by reacting equimolar amounts of bis(triphenylphosphoranylidene)ammonium chloride (BTPPACl, M = 574.03 g⋅mol^− 1^, Sigma-Aldrich) and sodium tetrakis[3,5-bis(trifluoromethyl)phenyl]borate (NaTPBF, M = 886.20 g⋅mol^− 1^, Sigma–Aldrich) in methanol: water mixture (2:1). The resulting precipitate bis(triphenylphosphoranylidene)ammonium tetrakis[3,5-bis(trifluoromethyl)phenyl) (BTPPATPBF) was isolated through filtration and was further recrystallized from acetone. Synthesized salt was dissolved in anisole (> 99%, Sigma-Aldrich) to obtain 5 mM solution further used as the organic phase. Poly(vinyl chloride) (PVC, Sigma-Aldrich) was used to increase the viscosity of the organic phase (organogel). Sodium chloride (M = 58.44 g⋅mol^− 1^, Fisher Chemicals) and tetrapropylammonium chloride (M = 221.81 g⋅mol^− 1^, Alfa Aesar) were used as received.

Artificial urine was a mixture of urea (150 mM), creatinine (50 mM), uric acid (1 mM), lactic acid (1 mM), citric acid (1.6 mM), and bilirubin (its concentration was the experimental variable) as the organic constituents. Inorganic salts included NaCl (90 mM), NH_4_Cl (25 mM), NaHCO_3_ (25 mM), NaH_2_PO_4_ (7 mM), MgSO_4_ (2.5 mM), Na_2_SO_4_ (10 mM), CaCl_2_ (2.5 mM), and FeSO_4_ (1 µM) [14]. Interfering species were all purchased from Sigma-Aldrich and cover dopamine (≥ 98%), paracetamol (≥ 99.0%) and ascorbic acid (≥ 99%).

Bilirubin (BIL, C_33_H_36_N_4_O_6_, M = 584.66 g⋅mol^− 1^, Sigma-Aldrich, ≥ 98%) was used as the analyte. The subsequent preparation of the BIL solution was conducted according to the procedure published in our recent work [14]. Briefly, the aqueous phase in the BIL electroanalytical study was a 10 mM Britton-Robinson solution, which contains equimolar amount of acetic acid (CH_3_COOH, M = 60.05 g⋅mol^− 1^, POCH), boric acid (H_3_BO_3,_ M = 61.83 g⋅mol^− 1^, POCH), phosphoric acid (H_3_PO_4_, M = 97.99 g⋅mol^− 1^, POCH) and sodium chloride. To adjust pH, the 1.0 M NaOH (M = 58.44 g⋅mol^− 1^, ChemPur) solution was used. The pH-meter (Orion STAR, A111, The Netherlands) was calibrated before each use with commercially purchased buffer solutions (Merck KGaA, 64271 Darmstadt Germany) with pH 4.01; 7.00, and 9.00 using a a polymer pH electrode (Polilyte Lab, Hamilton, Switzerland). A magnetic stirrer from Ohaus^®^ was utilized during the pH measurements. All water-based solutions were prepared using demineralized water (0.055 µS⋅cm⁻¹, Hydrolab^®^).

### Electrochemical cell 3D printing and characterisation

Capillary printing parameters are available in our recently published work [14]. Briefly, designs done in Tinkercad software were exported as.stl files, and were further cut into layers using PrusaSlicer. The.gcode files were imported to and printed with the Prusa Mini 3D printer. Printing parameters were fixed to ensure reproducibility, including layer height, speed, and temperature settings. For polyamide (PA) filament, key settings included a 0.15 mm layer height, 10–30 mm⋅s^− 1^ speed range, 215 °C, and 60 °C for nozzle and bed temperature, respectively. Other components (the cap with the apertures holding tube and electrodes) were printed using PLA filament. The final dimensions for all tubes were: height – 35 mm, outer diameter – 10 mm, and wall thickness – 2 mm. Tubes were printed using polyamide (PA) filament, exhibiting high resilience to organic solvents. Swelling tests were performed and no solvent uptake was observed up to studied 24 h period.

### Gelling procedure

**Scheme 1:**
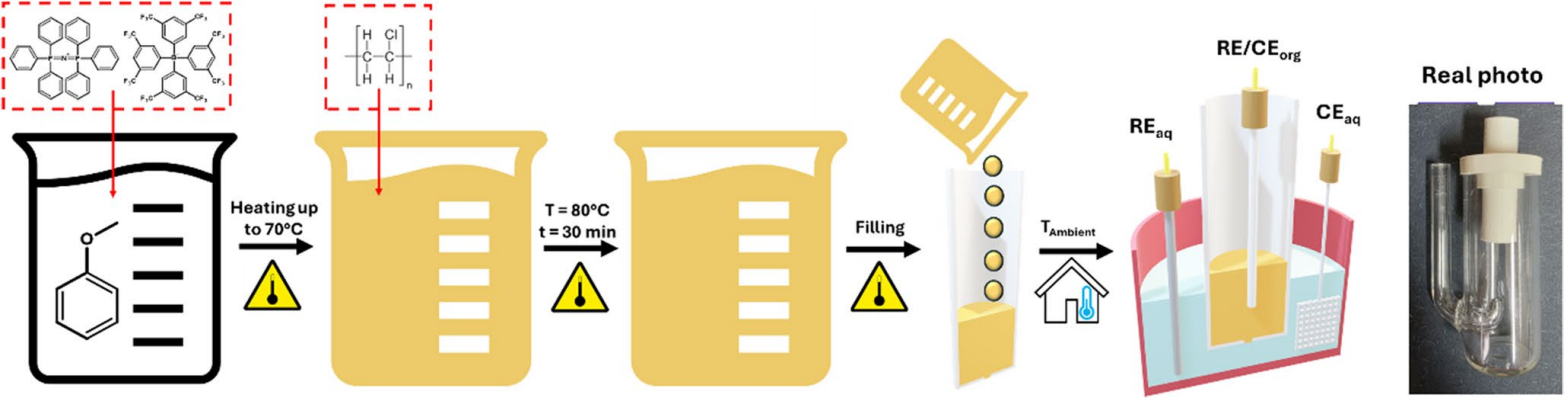
Step-by-step procedure depicting anisole-based gel placement into 3DP PA capillary. RE, CE, aq, and org stand for reference electrode, counter electrode, aqueous phase and organic phase respectively

To form the organic phase gel, we adopted the procedure published by A. Berduque et al. [51]. First, BTPPATPBF was dissolved in anisole placed in the volumetric flask to yield a 5 mM solution. Obtained solution was placed into a beaker situated on a heating mantel and water bath. Solution was heating to 70 °C. Next, PVC was added to reach a concentration of 10% w/v, keeping the temperature at the same level. Addition of PVC to cold organic phase will trigger aggregation and non-homogenous dispersion will be obtained. The temperature was then gradually increased to about 80 °C for 30 min. Stirring continued at this temperature until a homogenous gel phase was obtained. After 5 min. of cooling, the formed gel was placed in the 3D printed tube, followed by ITIES formation (the tube with a gel was immersed in the electrochemical cell equipped with a Lugging capillary and filled with the aqueous phase solution). Temperature was measured continuously using a probe placed inside the beaker. The organogel formation simplified protocol (along with the capillary real photo) is shown in scheme 1. The capillaries filled with gel should be used freshly after preparation. Long time storing is not recommended as due to progressive organic solvent evaporation the organic phase electrolyte may crystalize giving additional resistance.

### Electrochemical experiments

All electrochemical experiments described in this work were performed in a four-electrode arrangement. The counter and reference electrodes used in the aqueous phase were Pt wire and Ag/AgCl electrode. In turn, silver wire covered with AgTPBF (via application of anodic potential in saturated solution of NaTPBF dissolved in water with 10% addition of MeOH) served as the counter and the reference electrode in the organic phase. The quaternary ammonium cation – TPrA^+^ – was used as the model ion to characterize the water || anisole and the water || anisole gel-based systems supported with the 3D printed tube. The electrochemical cells used for the TPrA^+^ electrochemistry (cell I) and the BIL^2−^ detection (cell II) systems are schematically depicted below. Two electroanalytical techniques were employed to study gelled LLI and interfacial behaviour of anisole, this is cyclic and linear sweep voltammetry. The scan rate was set to 10 mV·s^− 1^ unless otherwise stated. Other parameters (width of the potential window and accumulation time) are provided in the respective figure labels.

All voltammetric measurements and calibration points were obtained from three recorded voltammograms. Reported values are given as mean ± standard deviation (SD) with *n* = 3. Calibration curves were obtained by linear least-squares regression using Origin software. The reported uncertainty of the slope is the standard error of the slope. The standard error (SE_intercept_) of the intercept from the regression was used for limit of detection (LOD) and quantification (LOQ) calculations. LOD and LOQ were calculated as LOD = 3·SE_intercept_/S and LOQ = 10· SE_intercept_/S, where S is the calibration slope. 95% confidence intervals (95% CI) for regression parameters calculation (95% of confidence level for parameters set for the linear fit). Baseline (blank) subtraction was applied prior to calibration where stated. All statistical analyses and regressions were performed in Origin software.

Electrochemical cell I.

**Table:** 

Ag	AgCl	x µM TPrA^+^ in 10 mM NaCl (aqueous)	5 mM BTPPA^+^ TPBF^−^ in anisole (liquid or gel phase)	AgTPBF	Ag

Electrochemical cell II.

**Table:** 

Ag	AgCl	x µM BIL^2−^ in artificial or real urine, pH 11 (aqueous)	5 mM BTPPA^+^ TPBF^−^ in anisole (gel phase)	AgTPBF	Ag

### Instrumentation

#### 3D printing

All capillaries were printed using PrusaMini 3D printer with the PrusaSlicer used as the software for the.gcode preparation.

#### Electrochemical experiments

The hardware AUTOLAB-PGSTAT 128 N potentiostat–galvanostat manufactured by Metrohm Autolab B.V., The Netherlands was used to record all voltammograms. The NOVA 2.1 software was employed for data recording.

#### 3DP pore characterization

Surface analysis of 3D-printed capillaries was carried out using scanning electron microscopy (SEM). The images were acquired with a Phenom Pro G6 Desktop microscope (ThermoFisher Scientific, USA). An electron beam acceleration voltage of 10 kV was applied, and a combination of backscattered electron detector (BSD) and secondary electron detector (SED) in a 1:1 ratio was used for recording micrographs. A 3D optical profilometer (UP-3000, Rtec Instruments, USA) controlled by Lambda software was employed to inspect the roughness of the 3D printed pore and its surroundings. The measurements were performed using a 5 ×magnification lens in confocal mode. The resulting images were analyzed using MountainsMap Imaging Topography 9 software.

### Artificial and urine preparation

Artificial urine was prepared as described (see Sect. 2.1. for chemical composition) and aliquoted. Next, artificial urine pH was adjusted to pH = 11 (1 M NaOH) and it was directly placed in the electrochemical cell. Aliquot spiking was performed with the BIL stock solution. Real urine samples were only subjected to a pH change by mixing the sample with the 1 M NaOH solution. Next, known amount of BIL stock solution was added. For real urine samples neither centrifugation nor filtration was applied. All samples and the BIL stock solution was stored at 4 °C.

## Results and discussion

### 3D printed capillary characterization

**Fig. 1:**
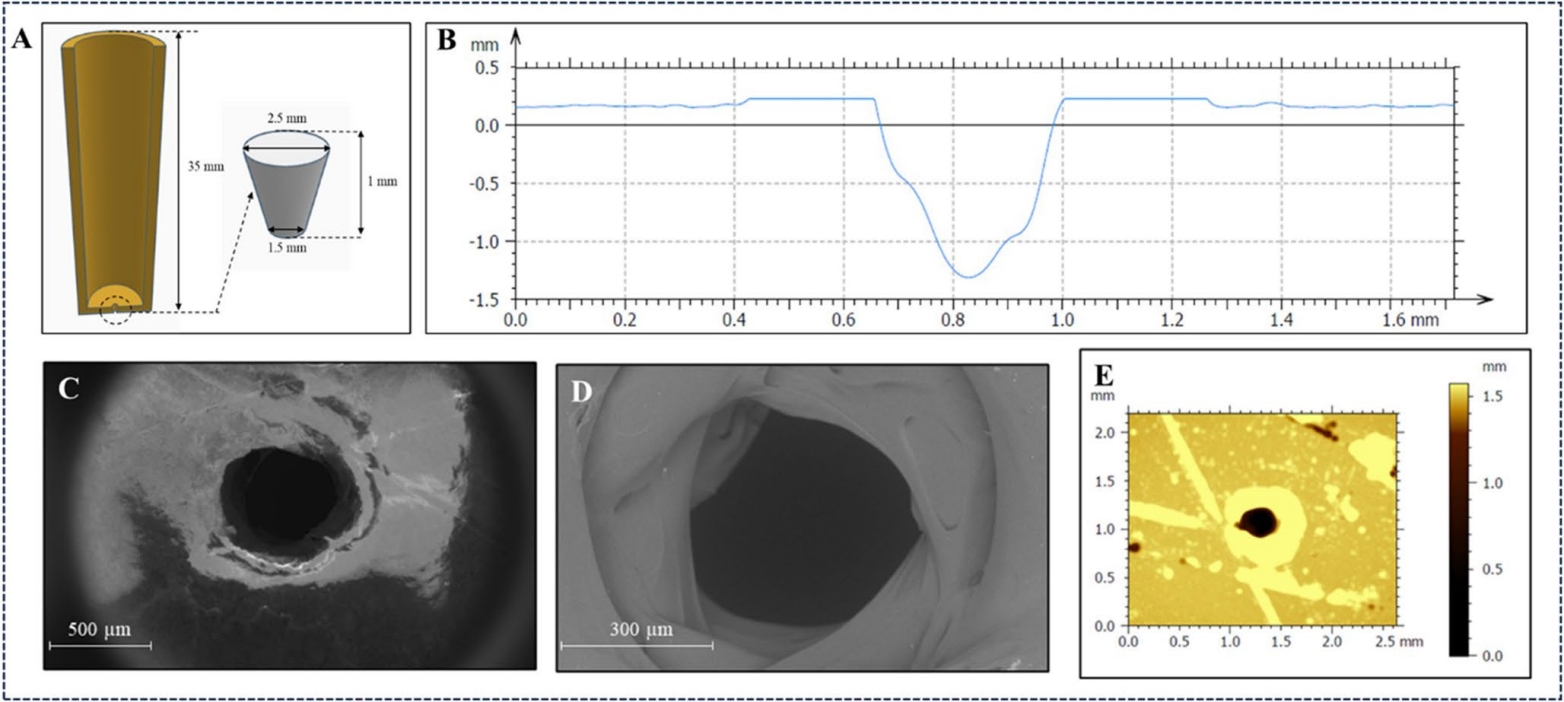
A – cross-section of the tube design; the inset on the right shows dimensions of the tapered hole in the bottom of the polyamide tube (drawing is not in scale); B – cross-section depth profile of the printed hole extracted from the optical profilometry analysis; C and D – SEM micrographs of 3D printed pore from the aqueous and organic point of view, respectively. E, F – images recorded with the optical profilometer of the pore side facing the aqueous phase during the measurement

Figure 1A illustrates the 3D-printed tube used as a support of the ITIES. The tube (capillary) has a pore at its base designed as a truncated cone (top circle diameter 2.5 mm; bottom circle diameter 1.5 mm; height 1.0 mm as shown in Fig. 1A). The application of this shape facilitated the printing process and allowed the prevention of filament overflowing and hence pore blocking. The diameters of the discs of the truncated cones were determined by SEM analysis and further inspected with optical profilometry. The results confirmed a truncated conical shape, with the interior pore diameter of 470 ± 15 μm (*n* = 4; side facing the organic phase) and the pore diameter of 330 ± 30 μm (*n* = 4; side facing the aqueous phase) – see Figs. 1B – F. We have also confirmed that the pore keeps its conical shape as the diameter of the entrance defined as the organic phase pore side (Fig. 1C) differs from the diameter of the pore exit, facing the aqueous phase side (Fig. 1D), yet significantly deviating from the dimensions set in design. The discrepancy between designed and obtained dimensions is a result of the first PA layer deposition, which upon extrusion enters the zone that was designed to be hollow and the filament overflowing for the following layers resulting from the heating nozzle aperture size (0.4 mm) and FDM printer resolution. The surface of the bottom of the capillary can be considered flat on the pore diameter scale (see Fig. 1E).

As indicated in the methodological section tubes were printed by FDM (Prusa Mini, 0.4 mm nozzle) with solvent-resistant PA to allow direct contact with anisole/PVC gel. FDM enabled rapid, low-cost prototyping and mechanical robustness but produced pore dimension deviations (~ 10% print to print) and microroughness (visible by SEM). For comparison, the alternative (relatively) low cost 3DP technologies are digital light processing (DLP) and stereolitogrpaghy printing (both resins based) that offers higher XY resolution and smoother pores. However, commonly available resins are more prone to organic-solvent attack and require post-processing. Micromachined glass/silicon pores provide superior pore dimensional control and surface smoothness but involve higher cost and limited design flexibility. In our application the PA-FDM route delivered the best compromise between solvent compatibility, mechanical stability, rapid iteration, and affordability, while maintaining acceptable reproducibility for electroanalytical performance.

### Electrochemical characterization of anisole || water liquid/liquid interface

**Fig. 2:**
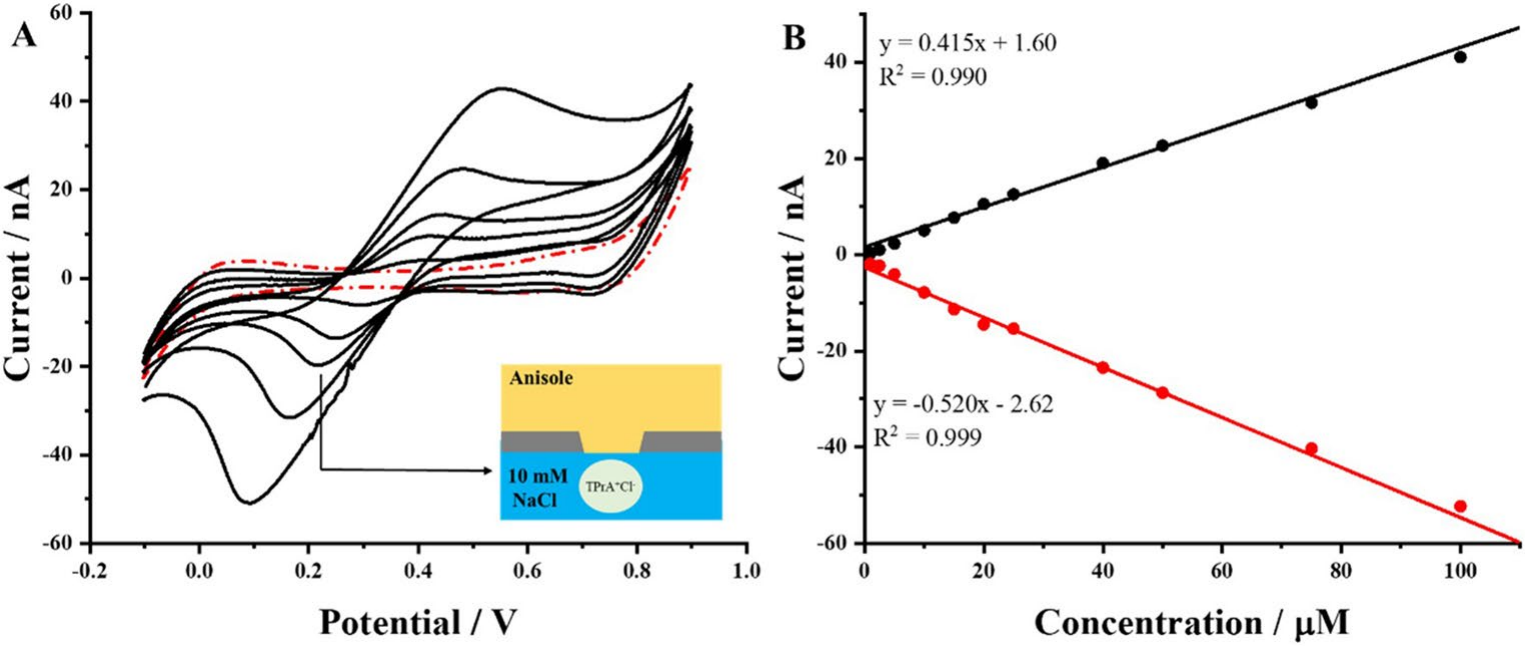
CVs recorded (**A**) in the absence (black solid line) and in the presence of 5; 15; 25; 50 and 100 µM TPrA^+^ (red dashed line) at the eLLI from the cell I (non-gelled organic phase). The inset shows a schematic representation of the pore supporting eLLI. (**B**) The calibration curve recorded for the positive and negative peak current attributed to the TPrA^+^ ion transfer plotted in function of its concentration (from 1 to 100 µM). The organic phase was supported by the 3D-printed PA tube. The scan rate was set to 10 m·Vs^− 1^

Initial characterization of the anisole-based ITIES was done with the ion transfer model probe, this is TPrA^+^ (accepted analogy to redox probes used for the characterization of the solid electrodes). Figure 2A shows selected cyclic voltammograms (CVs) recorded for a liquid organic phase system (see cell I). The black solid lines correspond to the presence of TPrA^+^ initially present in the aqueous phase, while the red dashed line is recorded in its absence. The obtained peak to peak separation is large (e.g. 0.205 V for 25 µM TPrA^+^) and increase with the model ion concentration. It is most probably the uncompensated resistance originating from the ohmic drop in the organic phase. Moreover, the recorded data highlight the semi-reversible nature of the ion transfer process, with well-defined peaks appearing in both, forward ($\:{TPrA}_{aq\to\:org}^{+}$) and reverse ($\:{TPrA}_{org\to\:aq}^{+}$) scans, with the peak intensities’ ratio equal to around 0.8. The calibration curve presented as Fig. 2B demonstrates a linear relationship between the peak current and TPrA^+^ concentration over the range of 1–100 µM. This linearity confirms the system’s capability for quantitative ion detection and provides sensitivity, linearity range, and detection limits similar to what was reported when other organic solvents (especially 1, 2-dichloroethane) were used for the organic phase preparation [39, 52, 53]. The slopes of the calibration curves for the positive and negative peak currents shown in Fig. 2B were found to be 0.415 ± 0.013 nA·µM^− 1^ for the positive and − 0.520 ± 0.016 nA·µM^− 1^ for the negative signals. This indicates that the transfer of TPrA^+^ from the organic to the aqueous phase always provides a higher portion of the Faradaic current (the ratio between forward and reverse slope is equal to 0.80). The viscosity of water (1.00 mPa·s at 20 °C) [54] and anisole (1.07 mPa·s at 20 °C) [55] are very similar. TPrA^+^ diffusion coefficient should be slightly lower for the latter solvent and the eventual expectation would be to observe lower overall values of the negative peak currents. Yet, the opposite is found as the absolute value of slope of the calibration curve plotted based on the analysis of the negative peak currents increased by around 20%. Since we did not observe a lower intensity of the Faradaic currents attributed to the TPrA^+^ transfer from the organic to the aqueous phase (see Fig. 2), interfacial preconcentration process is possible and may be related to slightly higher viscosity of anisole itself and the internal geometry of the pore (see Fig. 1D) [56]. The existence of wrinkles inside the pore created during the printing process will impede diffusion of the analyte to the volume of the organic phase. To further meet the requirements for the stripping analysis at the eLLI, the PVC was dissolved in anisole to increase its viscosity and lower the diffusion of the analyte after transfer from the aqueous to the organic phase [56].

### Characterization of gelled anisole/water liquid/liquid interface

**Fig. 3:**
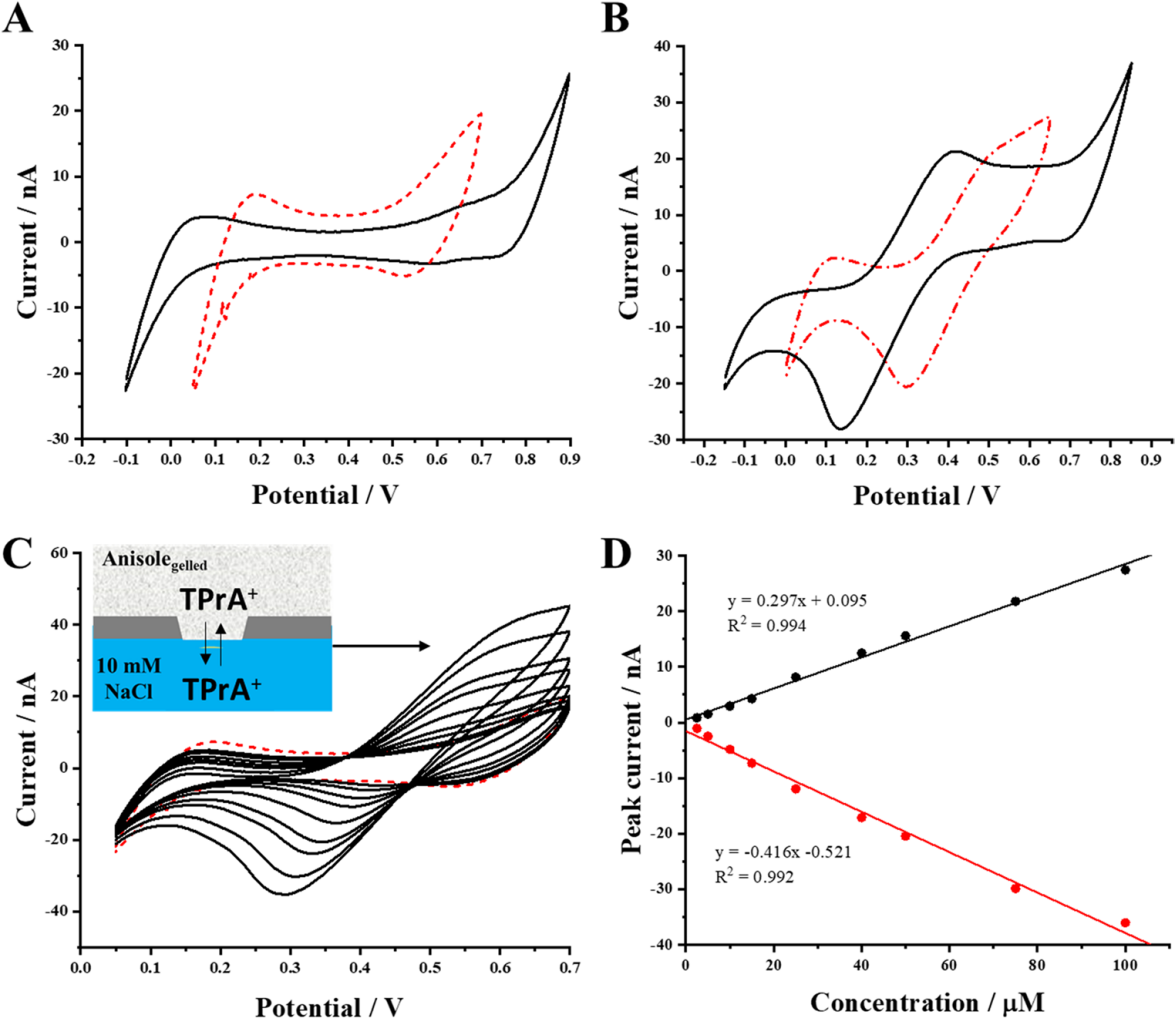
CVs recorded before (solid, black line) and after (red, dash-dot line) organic phase gelation (**A**) shows the blank readings while (**B**) was recorded after [TPrA^+^] = 50 µM addition. (**C**) CVs recorded at a miniaturized water/anisole-PVC gel interface for the increasing concentrations of TPrA^+^ (2.5–100.0 µM) initially dissolved in the aqueous phase (see cell II). The dashed red line is attributed to the blank (absence of TPrA^+^) reading. (**D**) The calibration curves plotted based on the CVs analysis from part (**C**). The scan rate was 10 mV⋅s^− 1^

Figure 3A compares the blank readings while Fig. 3B compares CVs recorded for 40 µM TPrA^+^ at the eLLI, using a liquid (black line) and a gelled (red line) organic phase, respectively. The gelation of the organic phase resulted in a noticeable increase of the capacitive currents and shrink the available width of the polarized part of the potential window. This means that the addition of PVC to the anisole changed the interfacial dielectric properties and affected the transfer of the background electrolyte ions, by lowering their formal ion transfer Galvani potential difference. Also, we have observed the drop of the positive current (related to TPrA^+^ transfer from the aqueous to the organic phase) from 19.03 ± 0.01 µA (liquid organic phase) to 12.59 ± 0.07 µA (gelled organic phase). In Fig. 3C we showed the CVs recorded for the TPrA^+^ contraction range from 2.5 to 100 µM added to the aqueous phase contacted with the gelled anisole placed in the 3D printed pore. The first concentration for which we were able to notice an additional Faradaic current different from a blank reading was equal to 2.5 µM. The gelation process affected the voltammetric features as (i) the peak-to-peak potential difference separation increased as the [TPrA^+^] reached 100 µM; and (ii) overall current intensities recorded for the same TPrA^+^ concentrations without application of any preconcentration parameters (time, applied potential difference) were lower after organic phase gelation. Nevertheless, the signals that were attributed to the $\:{TPrA}_{aq\to\:org;\:org\to\:aq}^{+}$ could be differentiated from the blank reading and hence, served as the electroanalytical information. Figure 3D shows the calibration curve that was plotted in the concentration range from 2.5 µM to 100.0 µM based on the analysis of the data set from Fig. 3C. The calibration curve fitting equation slopes for forward and backward currents were determined to be 0.297 ± 0.007 nA·µM^− 1^ and − 0.416 ± 0.013 nA·µM^− 1^, respectively. The slope calculated for the positive signals dropped by 29%, while for negative signals by 20% as compared with the non-gelled system. The ratio of the positive and negative slope was calculated and is equal to 0.71. Observed differences between gelled and non-gelled system can be summarized as: drop in the negative peak current after gelation process was expected and is attributed to the increase of the viscosity of the organic phase (decreased diffusion coefficient). The drop of the positive current is not expected, and it may be related to stabilization of the LLI within the pore which may have different dimensions as compared with the non-gelled system.

Nevertheless, initial set of data proved that voltammetry can be applied for the electroanalytical studies involving gelled anisole phase in the eLLI configuration. The correlation coefficient (*R*^*2*^) values for calibration curves derived from CV measurements indicate good linearity in the concentration range studied. The *R*^*2*^ values from Fig. 3C were 0.994 for positive currents and 0.992 for negative currents, further confirming the electroanalytical utility of the proposed methodology. Additionally, we have calculated and summarized the LOD, LOQ, and voltammetric sensitivity values, which are summarized in Table 1.

**Table 1:** Summary and comparison of electroanalytical parameters obtained for the TPrA^+^ detection at the water/anisole and water/gelled-anisole LLI

Analyte (sample)	Non-gelled/Gelled	Method	LOD/µM*	LOQ/µM**	1 st [Analyte]/µM***	Sensitivity/[nA·µM^− 1^]
TPrA^+^	Non-gelled	CV	(+) 3.46 (-) 2.87	(+) 11.5 (-) 9.58	2.50	(+) 0.415 (-) 0.520
TPrA^+^	Gelled	CV	(+) 4.13 (-) 4.16	(+) 13.78 (-) 13.89	2.50	(+) 0.297 (-) 0.416
TPrA^+^	Gelled	sLSV	(-) 0.43	(-) 1.44	0.50	(-) 0.428

LOD and LOQ were calculated according to the formulas: *LOD = 3xSD/S where SD is the error of the intercept and S is the voltammetric sensitivity and **LOQ = 10xSD/S where SD is the error of the intercept and S is the voltammetric sensitivity. *** This parameter stands for the first concentration that could be differentiated from the blank reading. sLSV stand for stripping linear sweep voltammetry. (+) – positive currents analysis; (-) – negative current analysis

### Stripping voltammetry at gelled µITIES – optimization study

**Fig. 4:**
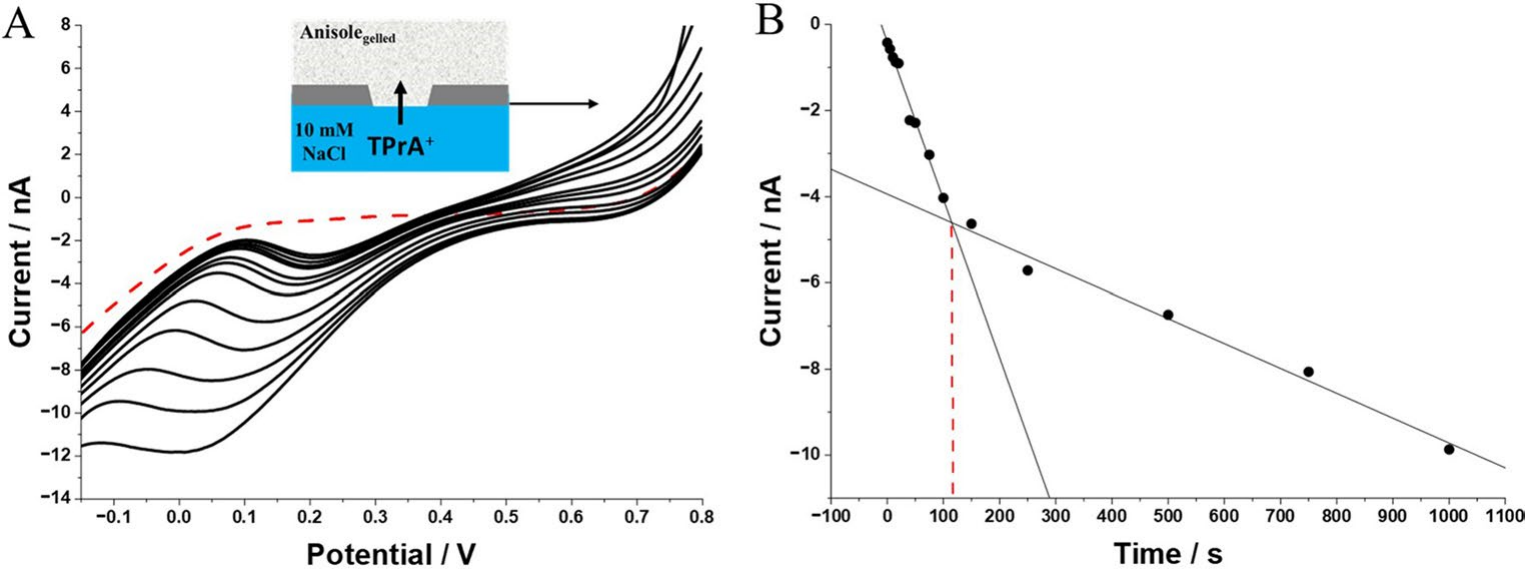
(**A**) LSVs recorded at a miniaturized water/anisole-PVC gel interface showing the effect of stripping time on the voltammetric peak current for 25 µM TPrA^+^ in 10 mM NaCl solution recorded with linear sweep voltammetry (LSV). (**B**) shows the dependence of the peak current plotted versus the preconcentration time Scan rate: 10 mV·s^− 1^. The polarization direction during LSV measurements was from higher to lower potential difference values. Potential difference applied during the preconcentration step was + 0.9 V

The gelation of the organic phase stabilizes the water || anisole interface and allows the ionic species preconcentration thanks to a thinner diffusion layer than in liquid organic solvent ($\:{\updelta\:}=\sqrt{Dt}$; $\:D\downarrow\:\:\to\:\:{\updelta\:}\downarrow\:$; originating from the lowered diffusion coefficient values) [57]. Our expectations, are that interfacial preconcentration will lead to lowered limits of detection and enhanced sensitivity. The preconcentration and stripping steps were studied with linear sweep voltammetry (LSV) which was used to polarize gelled ITIES. TPrA^+^, initially present in the aqueous phase, transfers towards the organic gel phase when the potential difference value exceeds + 0.35 V (see Fig. 3A). The TPrA^+^ transfers back towards the aqueous phase when the direction of polarization is reversed. The preconcentration potential of the cationic probes should be set at a value allowing TPrA^+^ transfer while avoiding Na^+^ transfer too (occurring at potential difference higher than 0.9 V). Next, we investigated the effect of the preconcentration time on the obtained peak currents. Figure 4A presents a series of LSVs recorded for varying preconcentration times ranging from 5 to 1000 s. Longer preconcentration time allows a greater accumulation of ions within the diffusion layer thickness on the organic phase side of the eLLI), resulting in higher peak currents recorded during the stripping step. Figure 4B shows the relationship plotted between the peak current and the preconcentration time. Two linear ranges can be distinguished. After reaching a certain point equal to around 100 s, the current values level off. Beyond 100 s, the rate of increase in peak current exhibits a marked reduction in slope, indicating the onset of saturation effects (and/or diffusion limitations caused by the extended $\:{\updelta\:}$ for the ions heading back to the aqueous phase). In other words, accumulation of analyte within the LLI approaches a limit, resulting in diminished responsiveness to extended preconcentration times. Based on the obtained data set, the time of preconcentration was chosen as 100 s (the compromise between signal intensity and the preconcentration time step).

**Fig. 5:**
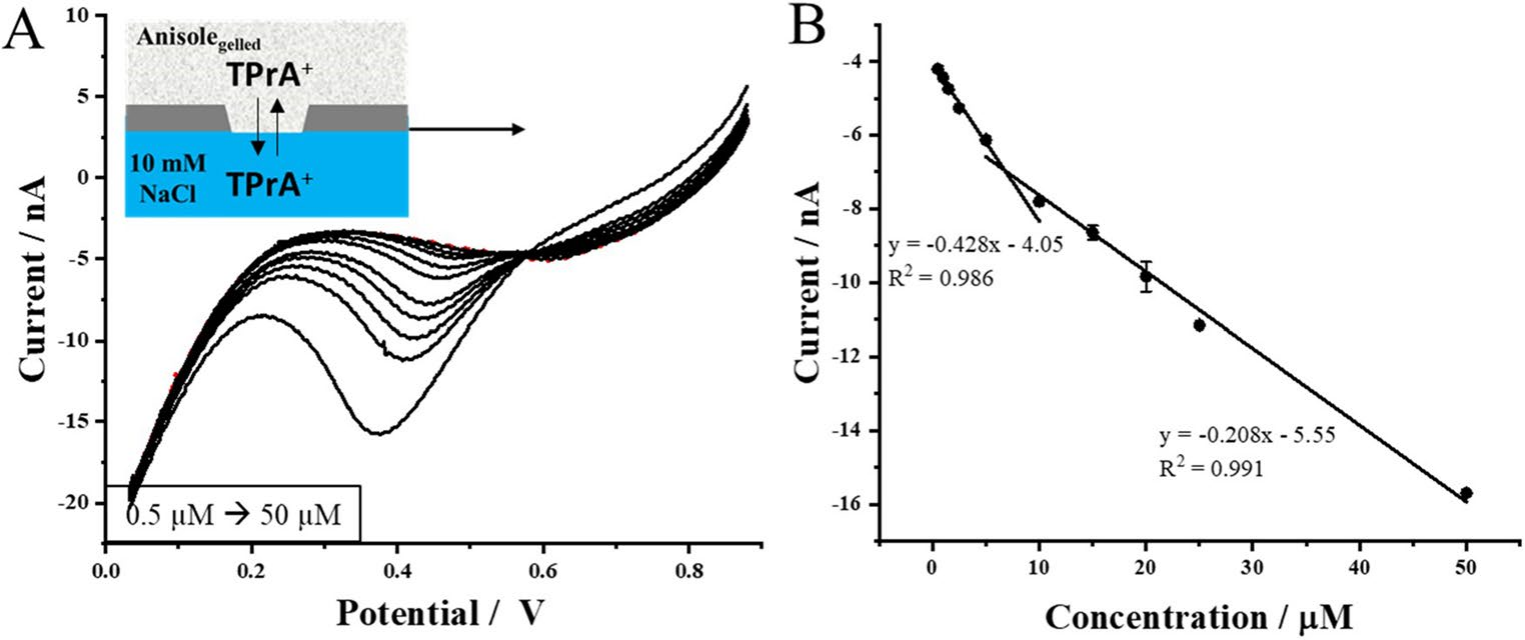
(**A**) LSVs recorded at a miniaturized water/anisole-PVC gel interface supported with 3D printed pore for various concentrations of TPrA^+^ (0.5–50 µM) with the applied stripping time of 100s. The dashed line is attributed to the blank (in the absence of TPrA^+^) reading. (**B**) The resulting calibration curve was plotted based on the LSVs analysis. The preconcentration potential for LSV was set to + 0.85 V. The direction of polarization was from higher to lower potential difference values

After optimizing the preconcentration time and defining the preconcentration potential, we have applied the selected parameters for the concentration-dependent study of the model ion TPrA^+^. As shown in Fig. 5A, a series of LSVs were recorded for increasing concentration of TPrA^+^, always initially dissolved in the aqueous phase, ranging from 0.5 to 50.0 µM. Prior to each recorded LSV a preconcentration time of 100 s was applied at a potential difference value being around 10 mV lower than the potential difference at which the limiting currents started rising (defined after recording a blank reading). For the given case, the applied potential difference was equal to + 0.85 V. Then, the scan was carried out with a polarization direction heading towards the lower potential values, ensuring that the ions accumulated within the LLI/diffusion layer on the organic phase side were stripped back to the aqueous phase. Interestingly, the calibration curve plotted based on LSVs analysis presents two linear dynamic ranges. Higher sensitivity was obtained for the lower concentration range (from 0.5 to 5 µM). The existence of the two linear ranges observed is primarily associated with the saturation of the adsorption sites at the water-gel interface and diffusion limitation within the gelled phase diffusion layer thickness. Another possibility is related to the organic phase electrolyte anion diffusivity (TPBF^−^) that is responsible for ensuring electroneutrality after TPrA^+^ transfer to the organic phase. By applying preconcentration, we were able to significantly lower the detection limit for the first measurable concentration. The lowest detectable concentration decreased from 2.5 to 0.5 µM. Also, the voltammetric sensitivity has increased by a factor of 1.53, for the comparison made for gelled-based systems with and without the application of preconcentration time, and taking into account that the first linear dynamic range after preconcentration time was applied. We have also calculated the LODs and LOQs which are summarized in Table 1, reaching sub-µM concentration levels only via simple preconcentration methodology. Also, this part of the work proves that similar electroanalytical output can be obtained with gelled anisole-based systems as compared with the works that rely on toxic 1,2-dichloroethane or 1,6-dichlorohexane [29, 58].

### Interference study and BIL^2-^ detection in artificial and real urine samples

We studied the effect of three chemical species with different physicochemical properties on the current intensity attributed to the BIL interfacial ion-transfer process. The analytes were dopamine (DOP), paracetamol (PAR), and ascorbic acid (AA). Chosen chemical species may exists in the urine matrix additional to its intrinsic parameters. Experiments were performed with a fixed [BIL] = 50 µM while known amounts of each potential interferent were added in the 1–500 µM range. Representative CVs and processed data showing the relative signal change are presented in Fig. S1 and Fig. S2, respectively. The interference of all three chemical species remained below 5% up to 100 µM concentration. At 500 µM, the signal attributed to BIL was unchanged in the presence of PAR and AA, whereas it decreased to about 75% in the presence of DOP. This is expected as PAR is non‑ionic and is therefore inactive at the ITIES whereas AA (being anionic molecules) undergoes ion transfer at potential difference values significantly lower compared to BIL. More interesting behavior was observed in the presence of DOP. Its addition to the aqueous phase caused a significant current variability (see Fig. S2) upon repetitive cycling at a single concentration, as reflected by large error bars. At pH 11 a small fraction of DOP can possess positive charge located within the amine group (p*K*_a_ ~ 9.5) meaning that formation of adducts with BIL is plausible, which may account for the observed effects. However, in real samples DOP concentrations in urine are low and typically do not exceed ~ 1.5 µM [59].

**Fig. 6:**
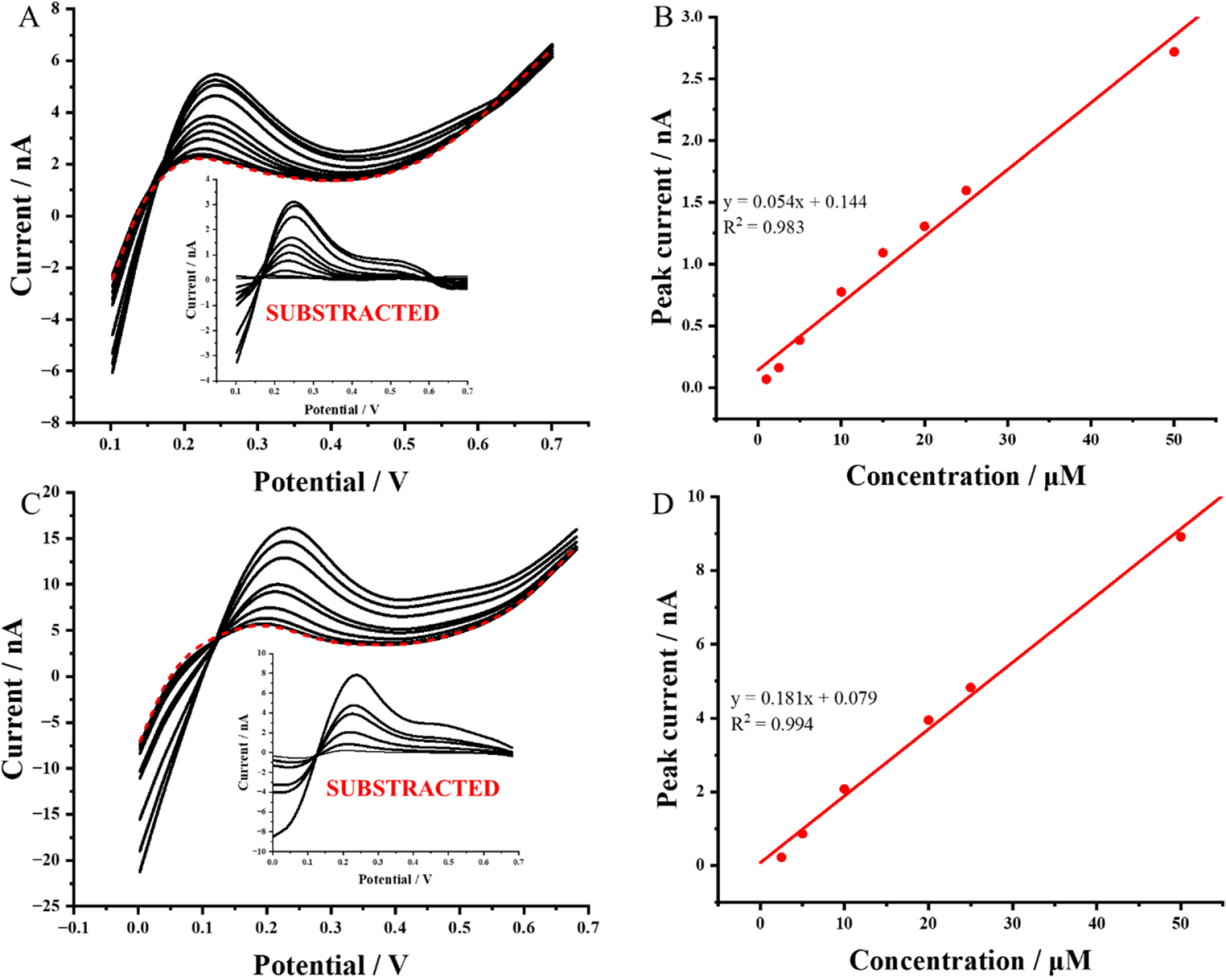
LSVs recorded at a miniaturized interface between artificial (**A**) and real (**C**) urine samples (the aqueous phase) and an anisole-PVC gel, supported by a 3D-printed pore, to detect increasing bilirubin concentrations. The preconcentration time was set to 20 s. Dotted lines indicate the baseline readings in the absence of BIL, while solid lines correspond to LSVs obtained after consecutive additions of BIL. Graphs **B** and **D** show the correlation between the obtained peak current and BIL concentration. The inserts present the LSV signals after background subtraction, clearly highlighting the voltammetric response at different BIL concentrations. The scan rate used for LSV was 10 mV⋅s⁻¹, with the preconcentration potential maintained at + 0.10 V for artificial and 0.0 V for natural urine. The polarization direction was from lower to higher potential values

BIL is a biologically significant molecule, a byproduct of heme catabolism that occurs primarily in the liver [49]. Elevated BIL levels are associated with conditions such as liver dysfunction, bile duct obstruction, and hemolytic disorders. Typically, BIL is measured in biological fluids like blood serum, but its presence in urine is particularly concerning. The detection of BIL in urine is a strong indicator of severe health issues, including liver diseases such as hepatitis or cirrhosis [60]. Given the clinical relevance of BIL, it is crucial to develop the rapid, cost-effective, and accessible analytical methods for its detection in biological samples, such as urine. To evaluate the applicability of the system developed in this work, we investigated the behavior of BIL^2−^ at the polarized water-gel interface in both artificial and natural urine samples. First of all, artificial urine was used. BIL^2−^ is an anionic analyte, which transfer from the aqueous to the gelled organic phase is observed when the potential difference is swept from the more positive to less positive potential difference values (in contrary to cationic TPrA^+^). Due to this reason, the preconcentration potential was set in the range from 0.0 V to + 0.1 V (it was adjusted based on the intensity of the limiting currents, that were kept below 20 µA, lower potential values may trigger the transfer of the background electrolyte ions to neighboring phases – Cl^−^ transfer to the organic phase and BTTPA^+^ transfer to the aqueous phase), while stripping was always performed via voltammetric scan towards more positive potential difference direction. As indicated in cell II, the aqueous phase was either artificial or real urine samples with a pH adjusted to the value of 11. In our previous work, we have found, that this pH assured the highest possible fraction of the anionic BIL^2−^ and gave optimized electroanalytical parameters [14]. Further increase in pH limits the potential window within which the eLLI is polarized as the concentration of the background electrolyte ions (particularly OH^−^) is increasing significantly. The obtained results are presented in Fig. 6A, showing a series of LSVs recorded for increasing concentrations of BIL^2−^ in the aqueous phase, represented by an artificial urine solution adjusted to pH 11 (cell II). The red dashed line corresponds to the baseline measurement for the system without BIL^2−^, while the black solid lines represent the voltammograms obtained after each addition of a standard BIL^2−^ solution in the concentration range of 1 to 50 µM. For each measurement, a preconcentration step of 20 s was applied to accelerate the experiment without compromising the detection limits. Figure 6B shows the corresponding calibration curve (Fig. 6A insert shows the background subtracted curves that were analyzed to plot calibration curves), which demonstrates linearity across the entire tested concentration range. The sensitivity of the system, defined as the slope of the calibration curve, was determined to be 0.054 ± 0.003 nA·µM^− 1^. To assess the applicability of the method in potential real-life applications, the artificial urine sample was replaced with natural urine, serving as the aqueous phase. The LSV with 20 s preconcentration time was applied to determine BIL at gelled-liquid interface supported within 3D printed polyamide pore. In the case of natural urine, the first detectable BIL concentration was 2.5 µM, which is lower than the 17 µM cut-off value [60]. Despite the complexity of natural urine as a matrix, the system demonstrated robust performance, achieving a calibration curve with a slope of 0.181 ± 0.007 nA·µM^− 1^. This result confirms the capability of the method to selectively detect BIL under these conditions, highlighting its potential for practical applications in clinical analysis. For the summary of electroanalytical parameters obtained for BIL^2−^ detection in spiked artificial and real urine samples please refer to Table 2. Obtained values of concentration for two detection limits are lower than 17 µM set as the BIL cut off level (this compound for healthy patients should be absent in this body fluid), confirming method suitability for such analysis [61]. It also should be noted that utilization of the 3D printing process results in the fabrication of tubes having pores with diameter ranging by ~ 10% (we are on the limit of printing resolution for the given dimensions). Correspondingly, the most suitable method for eventual BIL detection should be based on standard addition methodology. With this in mind, we have spiked the real urine sample to reach [BIL] = 5 µM and received 98.6% recovery value using standard addition method (see Fig. S3). Finaly, we compered the performance of the gelled anisole LLI supported with a 3DP tube with other available electrochemical BIL detection methodologies focused on urine as a real sample. The overview given as Table S1 indicates very good and comparable performance with the available state of the art.

**Table 2:** Summary and comparison of electroanalytical parameters obtained for bilirubin (BIL) detection at water/gelled-anisole LLI

Analyte (sample)	Non-gelled/Gelled	Method	LOD/µM*	LOQ/µM**	1 st [Analyte]/µM***	Sensitivity/[nA·µM^− 1^]
BIL (spiked artificial urine)	Gelled	sLSV	(-) 3.56	(-) 11.85	1.00	(-) 0.054
BIL (spiked real urine)	Gelled	sLSV	(-) 2.84	(-) 9.84	2.50	(-) 0.181

LOD and LOQ were calculated according to the formulas: *LOD = 3xSD/S where SD is the error of the intercept and S is the voltammetric sensitivity and **LOQ = 10xSD/S where SD is the error of the intercept and S is the voltammetric sensitivity. *** This parameter stands for the first concentration that could be differentiated from the blank reading. sLSV stand for stripping linear sweep voltammetry. (+) – positive currents analysis; (-) – negative current analysis

## Conclusion

In this study, we investigated the electrochemical behavior of bilirubin at the polarized liquid/liquid interface formed between anisole (both gelled and non-gelled) and an aqueous phase. In this respect, we have used 3D-printed polyamide tubes that served as the supports of the biphasic system. By dissolving polyvinyl chloride (PVC) in the anisole solvent, we increased its viscosity, leading to the formation of a gelled phase. Initial experiments using cyclic linear sweep voltammetry showed that current-potential dependencies in the presence of TPrA^+^ could be measured before and after the gelation of the organic phase. Through systematic optimization of preconcentration time and potential, we developed a stripping voltammetry procedure. The optimized parameters were then applied in concentration-dependent studies for TPrA^+^, demonstrating two distinct linear ranges in the calibration curves. Preconcentration improved detection limits, achieving 0.5 µM with LSV as compared to around 3 µM for non-gelled system (for TPrA^+^). Next, we applied this method to study bilirubin (BIL). Its behavior was studied in artificial and natural urine matrices. Despite the complexity of the urine matrix, we were able to detect BIL starting from concentrations equal to 1.0 µM (LOD = 1.54 µM) and 2.5 µM (LOD = 2.84 µM) for spiked artificial and real urine samples, respectively. Although the BIL should not be present in the healthy patient’s urine samples, the obtained LOQ is lower than its cut-off levels that for adults equals to 17 µM [61]. Overall, our aim was to prove that gelled anisole-water interface can be used for electroanalytical purposes, as confirmed in this work. What remains to be improved is reproducibility of the printed pore dimensions and further downscaling. Resin-based printing can achieve this, but better material compatibility with the organic solvents used for ITIES (or use of solvents compatible with existing printable resins) is required before resin-printed ITIES sensing platforms can be widely adopted.

## Supplementary Information

Below is the link to the electronic supplementary material.

Supplementary File 1 (DOCX 185 KB)

## Data Availability

Raw data published in this work are available under Zenodo repository; https://doi.org/10.5281/zenodo.14650747.

## Declarations

### Bioethical approval

Urine samples were analyzed in accordance with the decision of the bioethical commission No. 437/2023, dated December 13, 2023, issued by the Bioethics Committee at the Medical Center for Postgraduate Education. Urine donors provided their consent for collection and use of samples in this study.
