# Supplementary material for: Detection of bilirubin by stripping voltammetry at the gelled anisole – aqueous electrified interface supported with a 3D printed pore
Source: Mikrochim Acta. 2026 May 21;193(6):400. doi: 10.1007/s00604-026-08130-3 (PMC13194250; doi:10.1007/s00604-026-08130-3)
Supplement: Supplementary file 1 — (DOCX 185 KB) [file 604_2026_8130_MOESM1_ESM.docx]

**ELECTRONIC SUPPORTING INFORMATION**

**Detection of bilirubin by stripping voltammetry at the gelled anisole – aqueous electrified interface**

Karolina Marciniak,^a,b^ Konrad Rudnicki,^a^ Karolina Kowalewska,^a^ Grzegorz Kowalski,^a,c^ Michal Poltorak,^d^ Irena Walecka,^d^ Grégoire Herzog,^e^* Lukasz Poltorak^a^*

1. University of Lodz, Electrochemistry@Soft Interfaces Team, Department of Inorganic and Analytical Chemistry, Faculty of Chemistry, Tamka 12, 91-403 Lodz, Poland.
2. University of Lodz Doctoral School of Exact and Natural Sciences, Jana Matejki 21/23, Lodz, Poland
3. University of Lodz, BioMedChem Doctoral School of University of Lodz and Lodz Institutes of Polish Academy of Sciences, Jana Matejki 21/23, Lodz, Poland
4. The National Institute of Medicine of the Ministry of the Interior and Administration, Woloska 137, 02-507, Warsaw, Poland.
5. Université de Lorraine, CNRS, LCPME, F-54000 Nancy, France.

*Corresponding author: [lukasz.poltorak@chemia.uni.lodz.pl](mailto:lukasz.poltorak@chemia.uni.lodz.pl)

*Corresponding author: [gregoire.herzog@cnrs.fr](mailto:gregoire.herzog@cnrs.fr)

**Table of contents:**

Figure S1 – Ion transfer voltammograms recorded in the presence of the dopamine and interfering species – page 2.

Figure S2 – Effect of dopamine, ascorbic acid and paracetamol interference – page 2.

Figure S3 – Standard addition calibration curve – page 3.

Table S1 – Comparison of electroanalytical methods used for bilirubin detection – page 4.


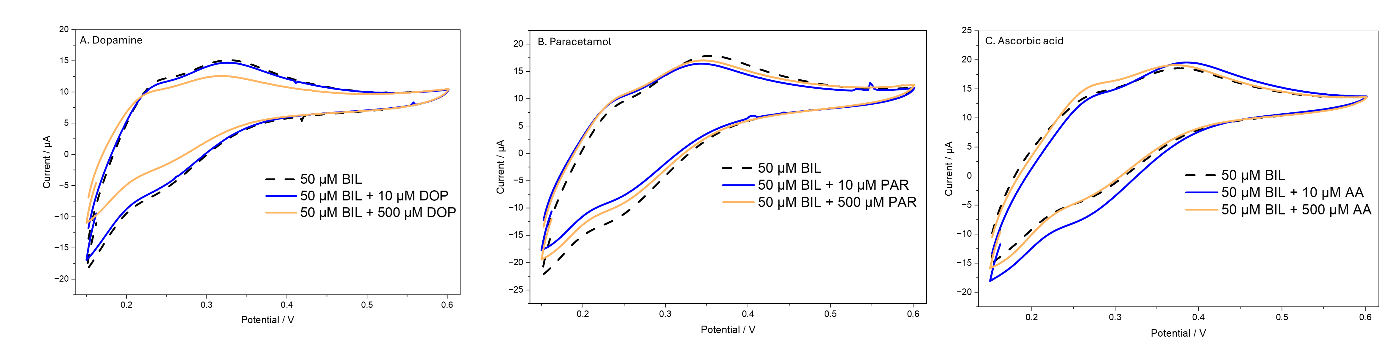


**Figure S1.** Ion transfer voltammograms recorded at the ITIES to study the effect of dopamine (A), paracetamol (B) and ascorbic acid (C) on bilirubin ion transfer signal. For the interfering species concetration please refer to the figure legend. The scan rate was 20 mV·s^-1^.


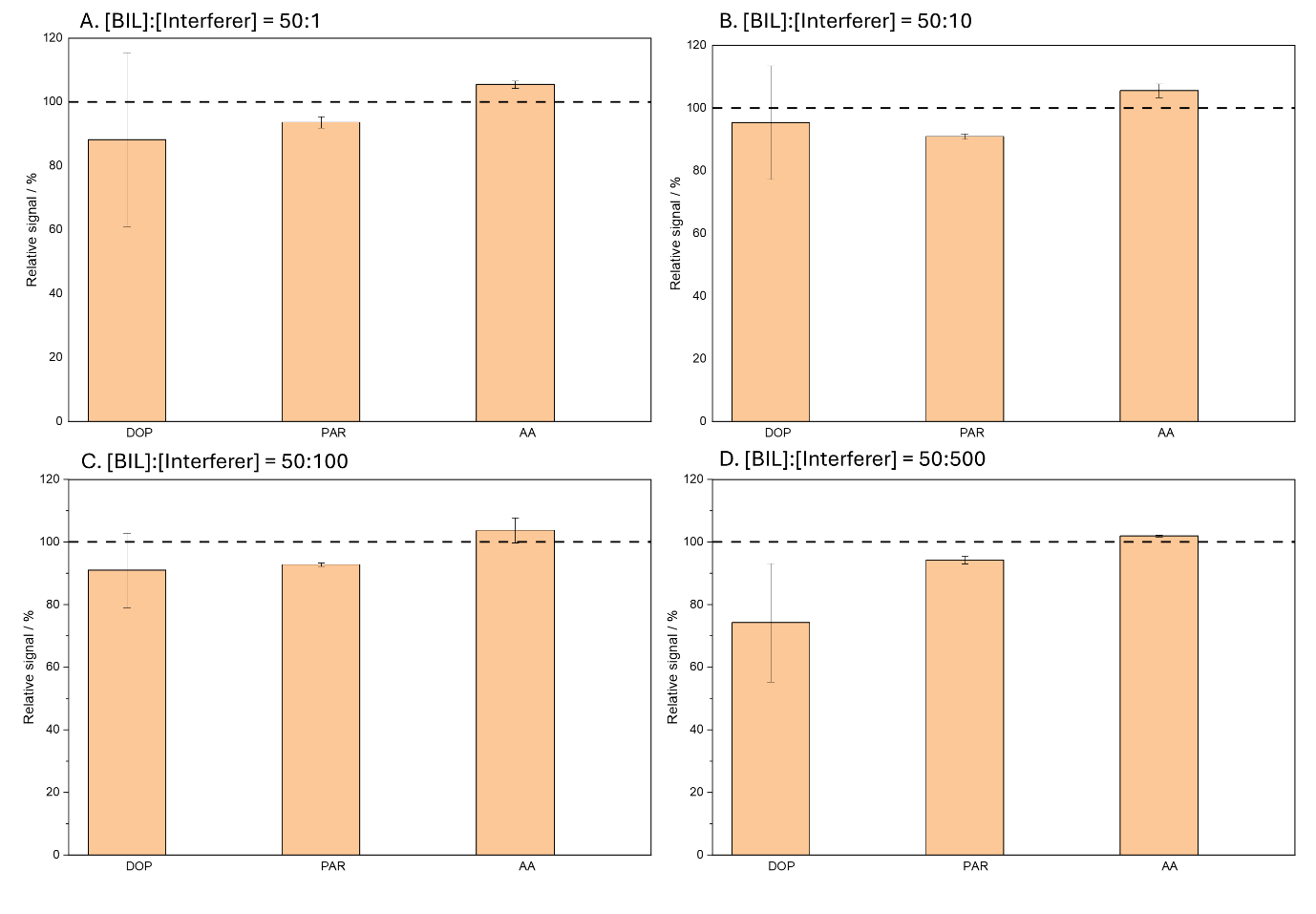


**Figure S2.** Relative signal change attributed to the [BIL] = 50 µM after addition of the dopamine (DOP), paracetamol (PAR) or ascorbic acid (AA) at different concentration levels: [BIL]:[Interfering species] are (A) 50 µM : 1 µM; (B) 50 µM : 10 µM; (C) 50 µM : 100 µM and (D) 50 µM : 500 µM.

**Figure S3.** Standard addition calibration curve prepared based on voltametric data set obtained at ITIES supported with the 3DP tube placed in an electrochemical cell containing real urine sample spiked with [BIL] = 5 µM.

**Table S1.** Overview of the BIL electrochemical detection methodologies validated on urine samples.

| **Sensing interface** | **Method** | **Linear dynamic range** | **LOD** | **References** |
| --- | --- | --- | --- | --- |
| **AgNPs@SPE** | CV and LSV | 1.7 – 15.4 µM | 1.7 µM | [1] |
| **Bilirubin Oxidase and Vanadium Nanozymes** | Amperometry | 1 – 26 µM | 1 nM* | [2] |
| **Nafion/rGO/GCE** | SWV | 2 – 20 µM | 0.84 µM | [3] |
| **BIL imprinted polymer** | CV | 1 – 100 µM | 0.75 µM | [4] |
| **CuBTC MOF SPE** |  | 5 - 130 µM | 0.36 µM | [5] |
| **Gelled anisole // aqueous interface** | CV | 2.5 – 50 µM | 2.84 µM** | This work |

*Calculated LOD is three orders of magnitude lower than the first concentration point on the linear dynamic range.

**LOD obtained directly in urine sample.
